# Short version of the Inventory of Parental Representations, a self-report for attachment assessment among adolescents

**DOI:** 10.1186/s12888-023-04704-0

**Published:** 2023-04-01

**Authors:** Marilou Lamourette, Fabienne Ligier, Francis Guillemin, Jonathan Epstein

**Affiliations:** 1 Pôle Universitaire de Psychiatrie de L’Enfant Et de L’Adolescent, Centre Psychothérapique de Nancy, 54521 Laxou, France; 2 Université de Lorraine, EA 4360 Apemac, Nancy, 54000 France; 3 PUPEA, 1, Rue du Dr Archambault, 54530 Laxou, France; 4 CHRU Nancy, Inserm, Université de Lorraine, CIC-1433 Épidémiologie Clinique, Nancy, 54000 France

**Keywords:** Adolescents, Reactive attachment disorder, Surveys and questionnaires, Inventory of parental representations

## Abstract

**Background:**

The Inventory of Parental Representations (IPR), a self-administered questionnaire, was developed primarily to identify styles of attachment in adolescence. However, it did not present stable psychometric properties in the various American studies carried out. The aim of this study was to adapt the IPR in French and to provide a shorter version with improved psychometric properties and sound content.

**Methods:**

The cross-cultural adaptation and content validity were carried out based on qualitative analysis by an Expert Committee and 10 non-clinical adolescents. For the quantitative analyses a cohort of 535 adolescent volunteers was enrolled, corresponding to 1070 responses, and divided into two groups: development and validation. The study of the metric properties of the adapted version of the IPR was realized in the development group, a sample of 275 responses. In case of mediocre results in the Confirmatory Factor Analysis, the development of a new and reduced IPR structure was planned using a mixed method including Classical Test Theory and Rasch Modelling in the development group. Subsequently, the study of the psychometric properties of the short, adapted version was confirmed in an independent sample of 795 responses (validation group).

**Results:**

Out of 62 items translated, 13 needed adaptations. The analysis of their metric properties produced mediocre results. Content and psychometric property analyses generated two Short version of the IPR in the development group: a paternal scale for Fathers (Short IPRF) with 15 items and a maternal scale for Mothers (Short IPRM) with 16 items. The sound content and good psychometric properties were confirmed in the validation group (Short IPRF: Comparative Fit Index = 0.987, Tucker-Lewis Index = 0.982, Root Mean Square Error of Approximation = 0.027; Short IPRM: Comparative Fit Index = 0.953, Trucker-Lewis Index = 0.927, Root Mean Square Error of Approximation = 0.068). Using Rasch modelling, the attachment was correctly measured overall especially for insecure attachment.

**Conclusions:**

A step-by-step process involving led to the generation of two questionnaires: a paternal scale, the Short IPRF, and a maternal scale with the Short IPRM providing opportunities to use this self-questionnaire to assess attachment among adolescents. Further work will provide a solid rating for this new tool.

**Supplementary Information:**

The online version contains supplementary material available at 10.1186/s12888-023-04704-0.

## Background

Attachment theory was defined by Bowlby in the 1960s [1]. The various styles of attachment were subsequently described by Ainsworth followed by Main through the Strange Situation Procedure (SSP) in children aged 12 to 24 months [2–4]. The Strange Situation Procedure is used to define several attachment patterns in children aged 12 to 14 months depending on their reactions when separated from the main attachment figure and in the presence of a stranger in progressively stressful situations. According to Ainsworth and Main, there are four early attachment and disorganized-insecure attachment. Secure attachment corresponds to a balance between the attachment system and the exploratory system. A secure child feels safe and confident in accessing his/her attachment figure during the SSP, and soon relaxes in the presence of his/her caregiver. The avoidant insecure attachment corresponds to hypo-activation of the attachment system. During the SSP, these children do not show signs of distress when separated from their caregiver and avoid or ignore the latter during reunion episodes. This avoidance reflects major anxiety on the part of the child due to inappropriate responses to their needs by those around them. The resistant-ambivalent insecure attachment corresponds to hyper-activation of the attachment system. These children seem pre-occupied with their caregiver during the SSP, alternating between seeking contact and resistance. The child therefore has little autonomy. The disorganized-disoriented attachment equates to the presentation of contradictory attitudes. This type of attachment is only mentioned incidentally in the SSP through the work conducted by Main. During this procedure, these children present disorganized or disoriented behavior in the parent’s presence and show indifference on separation. This type of attachment is more common if the parent has a psychiatric disorder or a history of psychological trauma is documented for the child [5].

Relationships with the primary attachment figures, mainly the parents, are reshaped during adolescence, heralding the process of separation and individuation. The adolescent develops his/her own caregiving system, transferring his/her attachment figures from parents to other people such as romantic partners. In adolescents, there are links between attachment disorders and mental health disorders [6–10]. Specific psychotherapies exist to treat attachment disorders [11, 12].

Two methods have been developed to evaluate attachment outside the SSP context: interviews and questionnaires. For adolescents, the interviews are mainly derived from the Adult Attachment Interview—an instrument developed for an adult population (George C, Kaplan N, Main M. Adult Attachment Interview Protocol. University of California, Berkeley. Unpublished Manuscript). These interviews require time and access to training. There is no Gold Standard for evaluating attachment in adolescence using self-administered questionnaires [13]. Several questionnaires can be used to assess attachment during adolescence. The Inventory of Parents and Peer Attachment is the questionnaire mostly used, even though the Inventory of Parents and Peer Attachment was not initially designed to measure attachment styles [14–16]. Indeed, the purpose of this scale is to assess the quality of the relationship with parents and peers, but it does not shed light on attachment style.

This suggests a need for additional questionnaires to assess attachment during adolescence. These questionnaires would have robust psychometric properties and would provide conclusive evidence in terms of attachment style. The Inventory of Parental Representations (IPR), a self-administered questionnaire, was developed in the US with the primary goal of identifying attachment styles as defined by Ainsworth and described above. The IPR authors have devised items to define the various insecure attachments with greater accuracy while taking specific adolescent characteristics into account. These items are based on qualitative interviews. Validation studies in the USA have used different self-administered questionnaires (depression, anxiety, etc.) and have shown a correlation with IPR sub-scores and with other self-administered questionnaire to assess attachment. However, the various IPR studies conducted in the US did not highlight stable psychometric properties [17–19]. Thus, according to the authors, it is possible to define secure or insecure attachment on the basis of the IPR. The generation of items was based on the different types of insecure attachment as defined by Ainsworth. The IPR was studied in English in an adolescent cohort [17].

The IPR has already been used in a French population without investigating its measurement properties [20]. A translation-back-translation process was implemented without transcultural adaptation, a step that is often deemed essential [21]. The use of an Expert Committee is important, particularly for content validity [22]. The lack of stability in the structure of the questionnaire suggested an element of uncertainty in terms of the content of the IPR and what it actually investigated. It was therefore relevant to re-examine the items included in the IPR and create a shorter questionnaire with a stable structure consistent with its content in an attempt to improve the psychometric properties of the scale [23]. The purpose of this work was to adapt the IPR into a French language version for adolescents between 13 and 18, and to propose a shorter version with improved psychometric properties and a clear content.

## Methods

### Participants

#### Sample

In this study, two sample cohorts were enrolled to cover both parts of the study, namely the qualitative part and the quantitative part. A sample cohort comprising 10 French adolescent volunteers between 13 and 18 years of age was enrolled for the qualitative part (sample 1) and participated in a semi-structured interview. The recruitment process was initiated by posting a notice in a medical establishment. Parents could then put forward their child to participate in the study. The informed consent of parents was obtained. All the interviews were conducted anonymously and no personal identifiable information was collected.

For the quantitative part, adolescents between 13 and 18 years of age were enrolled (sample cohort 2). The adolescents completed attachment assessment questionnaires including the IPR and their parents answered questionnaires focusing on socio-demographic data and lifestyle. The inclusion of adolescents was only effective if all the questionnaires sent to the parents were returned. All participants were volunteers and their responses were completely anonymous.

#### Ethical considerations

The parents’ informed consent was obtained for sample cohort 1. For sample cohort 2, parental consent was deemed to have been given when the adolescent and his/her parents completed all the questionnaires distributed. The study was completely anonymous, as agreed with the Ethics Committee. The French Ethics Committee validated this method of collecting consent. The protocol was approved by the Ethics Committee of the CHRU (Regional University Health Centre) in Nancy, France on 20/10/2016, as recommended by the authorities at the start of the study.

### Materials

The Inventory of Parental Representations (IPR) comprises two sections—one for the mother and one for the father—each including the same 62 items (Additional file 1). A distinction is made between maternal and paternal representations, even if the items are similar. The answers are based on a Likert scale ranging from 1 (Strongly disagree) to 4 (Strongly agree). The different versions of the IPR are described in Additional file 2: number of items, number of dimensions and the dimensions’ names. Studies conducted in the US do not make any IPR scoring recommendations.

In its original version, the IPR comprised seven dimensions, five of which specifically assessed insecure attachment [17]. The 7-dimensional structure was not found when a new exploratory factor analysis was carried out [18]. A study proposed a revised and shortened version of the IPR comprising 19 items divided into five dimensions. The author relied on a new exploratory factor analysis and interpretation of factor loadings to develop this version without any confirmatory analysis [19]. However, this structural instability is not explained.

### Procedure – Fig. 1-3

**Fig.1:**
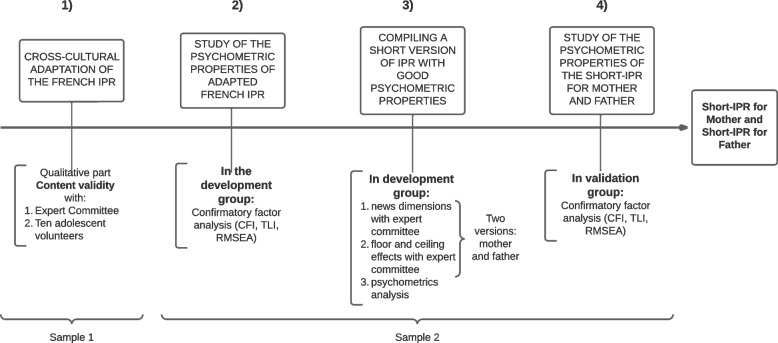
Methods of the development of the French version of the Short Inventory of Parental Representations – Study design

The study design is described in the Fig. 1. For this study, an Expert Committee was constituted, comprising four confirmed French-speaking child psychiatrists and a medical epidemiologist specialized in questionnaire adaptation. Three of the five Committee members were fluent in English. The whole Expert Committee analyzed the original version, while a smaller Committee, comprising two of the four confirmed French-speaking child psychiatrists and the medical epidemiologist, developed the IPR short version. This Expert Committee worked on the cross-cultural adaptation of the French IPR and devised a short version of the IPR.

#### Cross-cultural adaptation of the French IPR – qualitative part

**Fig. 2:**
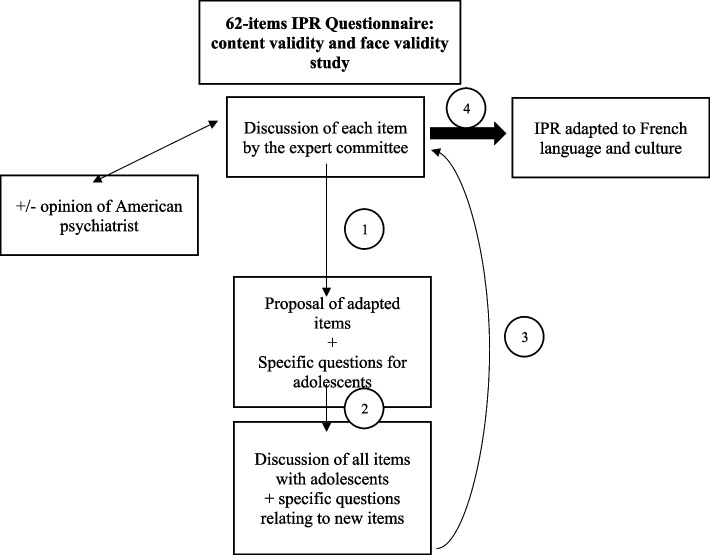
Process of Adapting the Inventory of Parental Representations (IPR) to French Language and Culture

This focused on content validity (Fig. 2). Good content validity is an essential property of a measurement scale, as it is an evaluation of the degree to which the content of the scale is relevant with respect to the construct it wants to measure and is recommended by the COSMIN group [24]. One of the components of content validity is face validity, which is how people perceive and comprehend items. The Expert Committee reviewed the French translation of each of the 62 items (box 1, Fig. 2). If any doubt was raised about the face validity of an item, it was discussed with adolescents (box 2, Fig. 2), and then considered by the Expert Committee. An English-speaking psychiatrist was contacted if the exact meaning of some items was unclear. Ten adolescent volunteers (sample 1) were individually interviewed on the face validity of the questionnaire. Ten participants were selected in order to saturate responses during the qualitative analysis and receive varied feedback. All 62 items were discussed and specific questions raised by the Expert Committee were put to them. A qualitative analysis of the responses was carried out. If at least two of the ten adolescents interviewed mentioned a problem with understanding an item, it was discussed again by the Expert Committee (box 3, Fig. 2). Based on these analyses and the attachment theory, the Expert Committee proposed a French cross-cultural adaptation of the IPR, used for the remainder of the study (box 4, Fig. 2).

#### Study of the metric properties of the adapted French version of the IPR – quantitative part

**Fig. 3:**
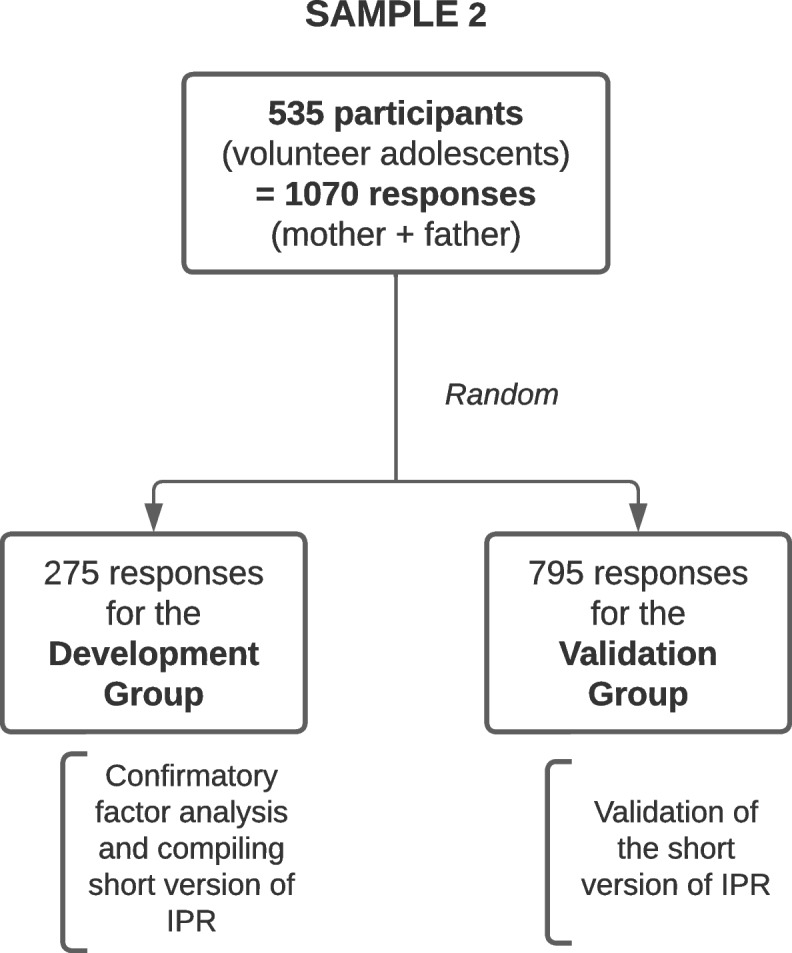
Constitution of the sample 2 for the study of the metric properties of the adapted French version of the Inventory of Parental Representations

The participants for the remainder of the study were taken from sample cohort 2 (Fig. 3). Two independent groups were randomized: one for an exploratory approach, further named the development group, constituted from adolescent volunteers from schools and colleges; one for a confirmatory approach, further named the validation group, constituted from school-enrolled adolescents and adopted adolescents.

COSMIN Group recommendations were followed in order to determine the number of subjects required for the psychometric analyses. Each group had to have a minimum number of four subjects by item, i.e., 62 × 4 = 248 responses to perform the analyses according to the recommendations of the COSMIN group [24]. The other responses have been used for the validation group to improve the analysis power.

Based on the classical test theory, confirmatory factor analyses (CFA) were carried out in the development group on the structures proposed by the developers to explore construct validity (Additional file 1).

#### Compiling a short version of the IPR – quantitative and qualitative part

In the case of mediocre results in the CFA of the existing structures, the development of a new, stable, content-based, IPR structure was planned according to the following steps in the development group. It was considered that the same structure might not be appropriate for the mother and the father.

- Step 1. New dimensions were identified from qualitative work carried out by the smaller committee. The 62 items were grouped into new dimensions consistent with the theory. Items or group of items deemed to be irrelevant for measuring attachment were removed and items which posed significant problems in terms of comprehension were also removed.
- Step 2. The floor and ceiling effects of responses to the items were taken into consideration in order to select discriminating items. If at least 80% of the sample size had the maximum or minimum response level for an item, it was discussed by the smaller committee and removed if it was not essential for measuring attachment styles.
- Step 3. Psychometric properties were analyzed using the development group data based on the structure proposed in step 1. In order to preserve the structure proposed in step 1, CFA were used iteratively in an innovating step-by-step approach. Between each CFA, the model was changed according to the modification indices of the model, reflecting the potential improvement of the model in the event of a change in the structure of the questionnaire and to align the consistency the new structure with the content. The aim was to establish a structure with good fit indices and factor loadings > 0.4. If the presence of an item or a group of items was unnecessary in terms of content, it was removed. If some items did not fit into a dimension or did not present properties acceptable in CFA but were important for assessing attachment in qualitative terms, the smaller committee could decide to retain them.

Analyses based on the Rasch item response model were conducted at the same time. If the Rasch analyses yielded unsatisfactory results, the dimension was discussed and modified again by the smaller committee. This process involved going back and forth between several CFA and Rasch analyses, combined with ongoing discussion focusing on content. The Short IPR obtained at the end of this process was used in the remainder of the study.

#### Confirmatory analyses of the Short IPR – quantitative part

The confirmatory analyses were performed on the validation group data. Based on the Short IPR structure, a CFA and Rasch analysis were carried out using the same criteria.

### Data analysis

In all the statistical analyses, as the items did not have the same positive or negative valence, the scores of certain items were inverted so that all the items followed the same direction in the analyses. A mixed statistical method was used to investigate IPR psychometric properties, based on both the classical test theory and the item response theory.

For analyses based on the classical test theory, two fit indices were used to assess the CFA fit: the *Comparative Fit Index* (CFI), Tucker-Lewis Index (TLI) and the *Root Mean Square Error of Approximation* (RMSEA). A CFA was considered to have a reasonable fit with the model if the CFI and TLI values were > 0.90 and if the RMSEA was < 0.08; the fit with the model was deemed good if the CFI and TLI ranged from 0.97 to 1.00 and if the RMSEA was ≤ 0.05 [25, 26]. Items presenting mediocre properties have factor loadings below 0.4 or modification indices that strongly linked the item with several other dimensions.

For the item response analyses, a single-parameter Rasch analysis was carried out. As the items were polytomous, a Rating Scale Model was used for the analysis [27]. Item response theory was used to check that latent trait was well covered by the items, particularly insecure attachment, and that the sequence of item response modalities was respected. Model fit was evaluated via the infit and outfit mean square (MNSQ). Significant MNSQ values less than 0.6 or greater than 1.4 were considered as a misfit to the model [28, 29]. The Person Separation Reliability index, which assesses internal consistency by measuring the discrimination capacity of the different response levels, was considered good if ≥ 0.80 [30]. All the analyses were carried out using R software version 3.5.1. [31]

## Results

### Cross-cultural adaptation of the French IPR

The Expert Committee reviewed the 62 items of the IPR. They discussed the translation of 30 items and decided to specifically interview adolescents about 9 items. The average age of the 10 adolescents interviewed on content validity was 15.1 years; there were 4 boys and 6 girls. No other sociodemographic data were collected as the latter are not required for this type of analysis [24]. None found the questionnaire disturbing or embarrassing. Three of them indicated that they would only give their questionnaire answers to health professionals because some questions were intimate.

All 62 items were reviewed again by the Expert Committee and a new adaptation of the IPR was proposed. Some items had to deviate further from the original English version in order to use a vocabulary that was more common, less complex and more adapted to adolescents. Item 35 “*Is competitive with me*” originally translated as “*Est en rivalité avec moi*” was finally adapted into: “*Entre en competition avec moi”/* “Competes with me”. Two adolescents did not understand “*rivalité*” (rivalry). It was preferable to move away from literal translation in order to keep the meaning of the expressions used in English. Item 54 "*Seems to be dependent and helpless*" originally translated as "*Semble être dépendant et sans défense*" was finally adapted into: "*J’ai l’impression que mon [parent] a besoin d’aide et qu’il/elle est sans défense*"/"*I feel that my [parent] needs help and that he/she is helpless*". In total, 13 items have been modified compared to the first French translation (Additional file 1).

### Study of metric properties of the French version of the IPR

Five hundred thirty-five adolescents responded to the questionnaires, for a total of 1070 responses; the responses concerning the mother and father were analyzed independently. The average age of the included adolescents was 14.3 years; 73.2% were female; 76.6% of parents were married. Sociodemographic data are presented in Table 1.

**Table 1:** Socio-Demographic Characteristics of study participants according to the group in which their responses were included: Development Group and Validation Group for the Study of the Psychometrics Properties of the Inventory of Parental Representations

Socio demographic characteristics	Total		Development Group		Validation Group	
	*N* = 1070		*N* = 275 (25.7%)		*N* = 795 (74.3%)	
	N(%)/Mean(SD)		N(%)/Mean(SD)		N(%)/Mean(SD)	
***NA***	*34*		*0*		*34*	
**Mean age (year)**	14.3	(1.50)	14.3	(1.49)	14.3	(1.58)
**Parent**						
Father	518	(50.0)	144	(52.4)	374	(49.1)
Mother	518	(50.0)	131	(47.6)	387	(50.9)
**Gender**						
Male	278	(26.8)	96	(34.9)	182	(23.9)
Female	758	(73.2)	179	(65.1)	579	(76.1)
**Family statut**						
In pairs	814	(81.4)	159	(61.9)	655	(88.)
Separated / divorced	138	(13.8)	75	(29.2)	63	(8.4)
Widow(er)	18	(1.8)	5	(1.9)	13	(1.7)
Single	30	(3.0)	18	(7.0)	12	(1.7)

*Legend*: Socio-Demographic Characteristics of Development Group and Validation Group used for the Study of the Psychometrics Properties of the Inventory of Parental Representations

The two groups were randomized. The development group included 275 responses (144 for fathers and 131 for mothers) and the validation group included 795 responses (374 for fathers, 387 for mothers and 34 without specified status). CFA results for the different versions of the original IPR conducted in the development group were not good. On the paternal and maternal sections of the seven dimensions of the IPR, CFA results were mediocre in the development group (paternal: CFI = 0.634, TLI = 0.618, RMSEA = 0.081; maternal: CFI = 0.539, TLI = 0.520, RMSEA = 0.090). For the revised 5-dimension version, the fit indices were reasonable only for the RMSEA; CFI and TLI were also mediocre (paternal: CFI = 0.846, TLI = 0.814, RMSEA = 0.075; maternal: CFI = 0.848, TLI = 0.817, RMSEA = 0.077). Given the mediocre properties, a new short version of IPR was constructed based on the content, with improved psychometric properties.

### Compiling a new version of the IPR

- *Step 1.* New dimensions were proposed by the smaller committee. Some items were removed from the scale due to ambiguities or comprehension problems raised in the content analysis. This applies to item 58: "*isn’t a strong person",* which defines the parent and not the relationship between adolescent and his/her parent. A dimension named *Concern* was initially proposed, which comprised items 2, 30, and 45. This dimension was completely removed because the items in this dimension measured the parent's personality and his/her fragility more than parental attachment relationship. The *Reliability* dimension was designed to measure the availability of the attachment figure and was therefore preferred. For some items, it was difficult to define the anticipated response according to attachment style, primarily because the central responses reflected secure attachment. These items, such as item 45: “*I worry about him dying*”, were removed. Six dimensions were retained in the Short IPR version: Reliability, Autonomy, Respect, Intrusion, Aggression and Availability.
- *Step 2.* Four items presented a floor/ceiling effect. E.g. item 13: "*likes to see me fail"* had a floor effect; 239 out of the 251 adolescents answered 1 in response to this item. The difficulty with this item appeared to be significant, but it was not required to evaluate the different attachment styles and was therefore removed.
- *Step 3.* The IPR properties remained mediocre when the responses given for both parents were analyzed simultaneously. Two versions of the IPR were therefore proposed, one for the father (Short IPRF) and one for the mother (Short IPRM), presented in Additional files 3 and 4, respectively. Some items presented mediocre properties and were therefore removed, such as item 24: "*places his needs first"*. Item 14: "*Doesn't approve my dating*" was also excluded. The IPR is aimed at adolescents from 13 years of age. Several adolescents did not respond to this item because they did not feel concerned by emotional relationships as yet. The *Aggression* dimension (items 17 and 35) was excluded from the analyses but retained in the Short IPRF and the Short IPRM. Performance for these items was mediocre, including a floor effect, but the content was deemed necessary for evaluating attachment, especially insecure attachment.

The final version of the Short IPRF comprised 15 items divided into six dimensions. CFA was carried out on 4 dimensions. The dimension assessing *Availability* had only one item, namely item 41 (*"My father does not respond when I'm in trouble"*) and could not be included in this analysis. However, it was decided to leave this item in the paternal scale to explore the representation of the father's availability to the adolescent, which is an important feature in assessing attachment. The Short IPRF items did not show any modification indices > 10 or any factor loadings < 0.4 during CFA in the development group.

The final version of the Short IPRM, relating to the mother, comprised 16 items divided into five dimensions. The *Reliability* dimension was not included in the maternal scale as the responses were not discriminatory. CFA was carried out using 4 dimensions. The Short IPRM showed only two items with a modification index > 10 and no item with a factor loading < 0.4 in the development group. Factor analysis for the final forms of both questionnaires gave good results with good or acceptable fit indices (Table 2).

**Table 2:** Results of Classical Test Theory Analyses of Short Paternal and Maternal Versions of the Inventory of Parental Representations for the study of the Psychometrics Properties of the Inventory of Parental Representations

	Short-IPRF		Short-IPRM	
	Development	Validation	Development	Validation
CFI	0.980^b^	0.987^b^	0.947^a^	0.943^a^
TLI	0.973^b^	0.982^b^	0.932^a^	0.927^a^
RMSEA	0.035^b^	0.027^b^	0.061^a^	0.068^a^
Number of items with loading < 0.4	0	1	0	0

*Legend*: Results of Classical Test Theory Analyses (Confirmatory Factor Analyses and Correlation Coefficient) of Short Paternal and Maternal Versions of the Inventory of Parental Representations Proposed in These Study (respectively Short-IPRF and Short-IPRM) in the Development Group and the Validation Group

CFI *Comparative Fit Index*, TLI *Tucker-Lewis Index,* RMSEA* Root Mean Square Error of Approximation*

^a^Reasonable adjustment

^b^Good adjustment

### Study of the metric properties of the Short IPRF and Short IPRM on the validation group

**Fig. 4:**
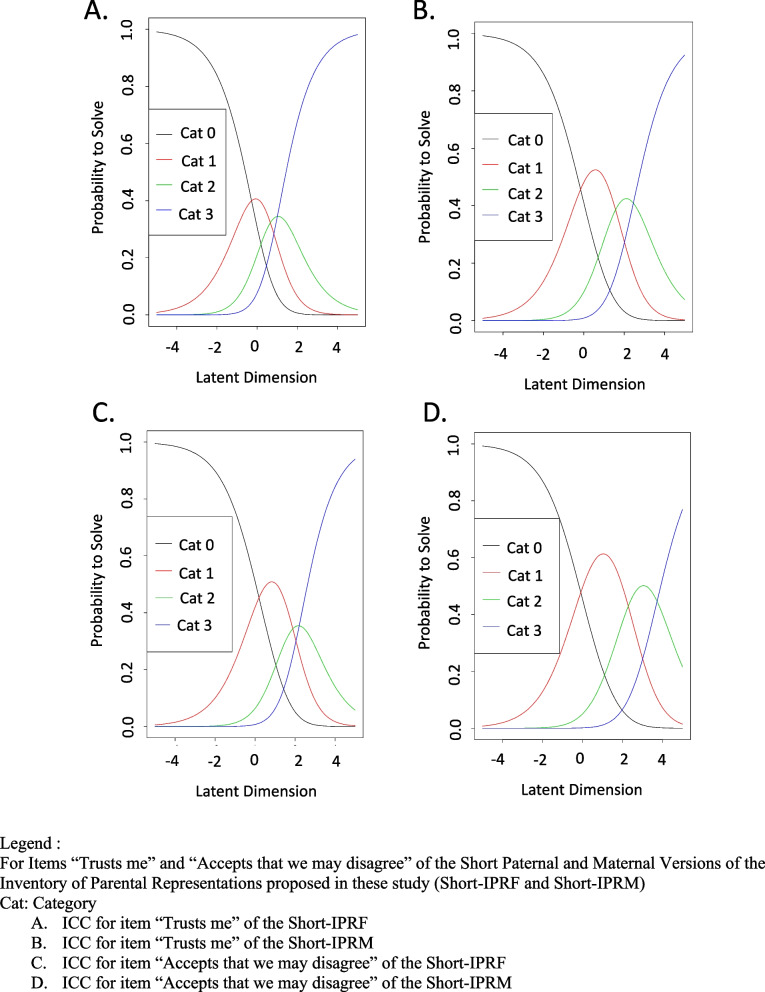
Item Response Category Characteristic Curves (ICC) for Fitted Item Response Theory Model in the Validation Group

The good results obtained with the fit indices in classical test theory were corroborated in confirmatory analyses: Short IPRF: CFI = 0.987, TLI = 0.982, RMSEA = 0.027; Short IPRM: CFI = 0.953, TLI = 0.927, RMSEA = 0.068 (Table 2). The adjustment was reasonable for the Short IPRM scale and good for the Short IPRF scale. Only 1 item, item 21 in the paternal version, presented a loading between 0.35 and 0.40 during validation group analyses. This item was retained in the short version after discussions with the smaller committee on the basis of preserving the content of the questionnaire. The sequencing of items in their dimension was good with the Rasch method (Fig. 4). Only one item presented a misfit in the Short IPRF, namely item 50, and none in the maternal scale. The Person Separation Reliability indices remained mediocre in each dimension studied in isolation (Table 3).

**Table 3:** Results of Item Response Theory Analyses for the Short Paternal and Maternal Versions of the Inventory of Parental Representations in the Validation Group

	**Dimension**	**Person Separation Reliability**	**MNSQ (fit)**
**Short-IPRF**	Dimension 1: Reliability	0.33	0
	Dimension 2: Autonomy	0.64	0
	Dimension 3: Respect	0.61	0
	Dimension 4: Intrusion	0.31	0
	Dimension 5: Aggression	*Excluded from the analyses*	
	Dimension 6: Availability	*No Rasch Modelling (only one item)*	
	**TOTAL**	**0.79**	**1**
**Short-IPRM**	Dimension 1: Reliability		
	Dimension 2: Autonomy	0.70	0
	Dimension 3: Respect	0.75	1
	Dimension 4: Intrusion	0.36	0
	Dimension 5: Aggression	*Excluded from the analyses*	
	Dimension 6: Availability	0.38	0
	**TOTAL**	**0.86**	**0**

*Legend*: Results of Item Response Theory Analyses for the Short Paternal and Maternal Versions of the Inventory of Parental Representations proposed in these study (Short-IPRF and Short-IPRM) in the Validation Group

## Discussion

The IPR is a self-administered questionnaire assessing attachment in adolescence with a specific focus on insecure attachment styles. However, none of the structures of the original and revised IPR versions was confirmed in the French population after the items were adapted. The psychometric properties of both these versions remained mediocre in the present study. These results could be anticipated given the fact that the structure was not stable in the various IPR studies, as shown by the difference in the number of dimensions identified during the various exploratory factor analyses. This can be partly explained by the methodology used by previous authors in elaborating the revised version of the IPR.

The IPR items are particularly interesting since they are based on the attachment styles described by Ainsworth and seek to portray insecure attachment more effectively [17]. Thus it was fitting to seek to improve the psychometric properties of this questionnaire. Through an iterative process of dimension generation, the psychometric properties were improved and a stable model was proposed. In the end, six dimensions were identified using a qualitative approach, based on attachment theory.

The structure of each dimension of the IPR original versions from Solow and Shapiro is presented in Additional file 1. The structure of each dimension of the Short-IPR is ultimately completely different from initial scale. To propose these new dimensions, the expert committee questioned the interest of each item for each dimension, according to the Bolwby, Ainsworth and Main attachment theory. For example, some items were deleted because they focused more on the condition of the parent than on the internal measure of the feeling of security, such as item “seems to be dependent and helpless” (item 54 of the original version – dimension Demanding/Disappointed Object). With these new dimensions, Short-IPR has stable and robust psychometric properties.

The Aggression dimension was excluded from the analyses. Item 17, for example: *"Does things to humiliate me"* was particularly difficult and had a floor effect. This partly explains why it was not possible to retain it in the final structure. However, Aggression can be present in the relationship between the adolescent and his/her parents in case of insecure attachment, especially resistant-ambivalent insecure attachment [32–34]. The committee therefore opted to keep it in both versions. A reliable scoring system should now be established.

It was clinically relevant to create two separate questionnaires based on paternal and maternal responses. Other questionnaires, such as the Inventory of Parents and Peer Attachment, distinguish between paternal and maternal responses based on the assumption that attachment style differs from early childhood, depending on the attachment figures [14, 35].

The item responses were all ordered according to the Rasch analysis. Qualitatively, items that seemed easier to the experts were indeed classified as easier in the Rasch analysis. The results show, for each dimension, that the latent trait was covered by the items predominantly at one end of the latent trait. This is the part of the scale that corresponds to insecure attachment where the questionnaire is probably the most discriminating. A lower discrimination parameter was evident for adolescents at the other end, corresponding to secure attachment. This explains why the Person Separation Reliability indices remained mediocre for most of the dimensions: the items’ coverage of insecure attachment meant a lower discrimination for secure attachment. Since the purpose of this scale is to define insecure attachment versus secure attachment and distinguish the various representations in terms of insecure attachment, it is particularly interesting to cover mainly insecure attachment. In fact, the encounter with adolescents in care is often done in an emergency context. It is important to establish a relationship with them quickly. It has been shown that individuals with insecure attachment have more difficulties during adolescence [36]. Adolescents with insecure attachment type preoccupied are more often in conflictual relationships. Distinguishing the type of insecure attachment in an adolescent allows for rapid adjustment of the therapeutic relationship.

Cultural differences between the USA and France may be one of the reasons why the structures proposed in the American versions of the IPR did not give good results in this study. However, during the cultural adaptation process, few items posed difficulties that could not be resolved and that involved deep changes in the items or the questionnaire. The 62-item version may not have good psychometric properties, regardless of language. Although attachment is universal, there may be cultural differences in attachment patterns. However, no differences have been proposed in the literature within Western cultures. For non-western cultures, the modalities of attachment assessment could be discussed and the fitness of the IPR reevaluated in the context of said cultures [37]. For the sake of completeness, it would be interesting to validate the proposed shorter structure in other western cultures and in English-speaking countries in particular. The expected results would be to find the same results than in the present study. If this shortened structure is indeed stable in different Western cultures, then the Short IPR could be used as a short, high-performance, international tool, the content of which is designed to measure attachment.

Short-IPR was developed by interviewing adolescents between 13 and 18 to allow a rapid assessment of the adolescent attachment style. Psychometric properties of this scale are encouraging for the use of this tool in everyday practice. But overall, it is necessary to determine the thresholds of the Short-IPR, by comparing its results with other attachment assessment tools, including interviews. It will be then interesting to test the finalized tool to a population of young adults (15–25) and to propose Short-IPR in clinical population.

## Conclusion

This study provides a new tool for assessing attachment among adolescents in the form of a self-questionnaire based on representations of parental relationships, taking differences between fathers and mothers into account. A step-by-step process involving both content analysis and psychometric property analysis based on the classical test theory and item response theory led to the generation of two questionnaires: a paternal scale, the Short IPRF (15 items), and a maternal scale, the Short IPRM (16 items). Their good psychometric properties were confirmed in an independent sample. This new structure should be tested and used in other languages and cultures, on English-speaking adolescents, in particular. This new tool adapted for the French population has good properties and is based on sound content. The work should be continued in order to obtain an accurate scale rating based on these stable properties.

## Supplementary Information

**Additional file 1. **Original version of the Inventory of Parental Representations, initial French translation and adapted French version. Presentation of the different versions (translation and adaptation) of the Inventory of Parental Representations (in French).

**Additional file 2. **Different versions of the Inventory of Parental Representations proposed by the authors. Table which present the different structures of the Inventory of Parental Representations depending on different studies.

**Additional file 3. **Paternal short version of the Inventory of Parental Representations: The Short IPRF. Presentation of the new questionnaire: The Short Inventory of Representations for Fathers (in French).

**Additional file 4. **Maternal short version of the Inventory of Parental Representations: The Short IPRM. Presentation of the new questionnaire: The Short Inventory of Representations for Mothers (in French).

## Data Availability

The datasets used and analyzed during the current study are available from the corresponding author on reasonable request.
