# Supplementary material for: Short version of the Inventory of Parental Representations, a self-report for attachment assessment among adolescents
Source: BMC Psychiatry. 2023 Apr 1;23:221. doi: 10.1186/s12888-023-04704-0 (PMC10068148; doi:10.1186/s12888-023-04704-0)
Supplement: Supplementary file 1 — Additional file 1. Original version of the Inventory of Parental Representations, initial French translation and adapted French version. Presentation of the different versions (translation and adaptation) of the Inventory of Parental Representations (in French). [file 12888_2023_4704_MOESM1_ESM.docx]

Additional file 1. Original version of the Inventory of Parental Representations, initial French translation and adapted French version.

| Item | **Original version** | **Initial French translation** | **Adapted French version** |
| --- | --- | --- | --- |
| 1 | doesn’t understand me | ne me comprend pas | ne me comprend pas |
| 2 | I worry about him | Je me fais du souci pour lui | Je me fais du souci pour mon père |
| 3 | is the best father of the world | est le meilleur père du monde | est le meilleur père du monde |
| 4 | helps me do the things I want to do | m'aide à faire les choses que je veux faire | m'aide à faire les choses que je veux faire |
| 5 | I am very much like my father* | Je suis vraiment comme mon père | Je suis vraiment comme mon père |
| 6 | lets me chose for myself | me laisse choisir pour moi-même | me laisse faire mes propres choix |
| 7 | we like the same things* | Nous aimons les mêmes choses | Nous aimons les mêmes choses |
| 8 | has too little confidence in me | a trop peu confiance en moi | a trop peu confiance en moi |
| 9 | expects me to be perfect | attend de moi que je sois parfait(e) | attend de moi que je sois parfait(e) |
| 10 | needs me | a besoin de moi | a besoin de moi |
| 11 | sacrifices a lot for me | sacrifie beaucoup de choses pour moi | sacrifie beaucoup de choses pour moi |
| 12 | is jealous and angry* | est jaloux et en colère | est jaloux et en colère par rapport à moi** |
| 13 | likes to see me fail | aime me voir échouer | aime me voir échouer |
| 14 | doesn't approve of my dating | n'approuve pas que je sorte avec des filles (garçons) | n'approuve pas mes petits copains / petites copines** |
| 15 | wishes I were perfect | souhaite que je sois parfait(e) | souhaiterait que je sois parfait(e)** |
| 16 | is quite self-centrered* | est plutôt égocentrique | est plutôt égocentrique (pense à lui avant les autres)** |
| 17 | does things to humiliate me | fait des choses pour m'humilier | fait des choses pour m'humilier |
| 18 | doesn't give me any space* | ne me laisse aucun espace | ne me laisse aucun espace |
| 19 | is too concerned with my intimate relationships | se préoccupe trop de mes relations intimes | se préoccupe trop de mes relations intimes |
| 20 | lets people take advantage of him* | laisse les gens profiter de lui | laisse les gens profiter de lui |
| 21 | is a pushover* | se laisse facilement avoir | se laisse marcher dessus |
| 22 | can be revengeful* | peut être "revanchard" | peut être rancunier** |
| 23 | we have a perfect understanding* | Nous avons une parfaite compréhension mutuelle | Nous nous comprenons parfaitement** |
| 24 | places his needs first* | fait passer ses besoins en premier | fait passer ses besoins en premier |
| 25 | is disappointed in me | Je le déçois | Je le déçois |
| 26 | thinks he is always right | pense qu'il a toujours raison | pense qu'il a toujours raison |
| 27 | is too protective of me* | me protège trop | me protège trop |
| 28 | can’t stand up to me | ne peut pas me tenir tête | ne peut pas me tenir tête |
| 29 | I can easily take advantage of him* | Je peux facilement profiter de lui | Je peux facilement profiter de lui |
| 30 | I feel protective of my father | Je veux protéger mon père | J'ai l'impression de devoir protéger mon père** |
| 31 | makes me feel special | me donne l'impression d'être unique au monde | me donne l'impression d'être unique** |
| 32 | doesn't give me any privacy* | ne me laisse aucune intimité | ne me laisse aucune intimité |
| 33 | I want him to be proud of me* | Je veux qu'il soit fier de moi | Je veux qu'il soit fier de moi |
| 34 | I hope I can fulfill his dreams for me* | J'espère pouvoir réaliser les rêves qu'il a pour moi | J'espère pouvoir réaliser les rêves qu'il a pour moi |
| 35 | is competitive with me | est en rivalité avec moi | entre en compétition avec moi** |
| 36 | is more interested in his work than her family | est plus intéressé par son travail que par sa famille | est plus intéressé par son travail que par sa famille |
| 37 | is always there when I need him | est toujours là quand j'ai besoin de lui | est toujours là quand j'ai besoin de lui |
| 38 | I wish I could make him happy* | J'aimerais pouvoir le rendre heureux | J'aimerais pouvoir le rendre heureux |
| 39 | wants me to be independent | veut que je sois indépendant(e) | veut que je sois indépendant(e) |
| 40 | I hate to let him down* | Je déteste le décevoir | Je déteste le décevoir |
| 41 | doesn't react when I’m in trouble | ne réagit pas quand j'ai des ennuis | ne réagit pas quand j'ai des ennuis |
| 42 | is not the kind of person I want to be | n'est pas le genre de personne que je veux être | n'est pas le genre de personne que je veux être |
| 43 | accepts my decisions | accepte mes décisions | accepte mes décisions |
| 44 | trusts me | me fait confiance | me fait confiance |
| 45 | I worry about him dying* | L'idée qu'il puisse mourir m'inquiète | L'idée qu'il puisse mourir m'inquiète |
| 46 | can't stop me | ne peut pas m'arrêter | ne peut pas m'arrêter |
| 47 | likes me to stay home* | aime que je reste à la maison | préfère que je reste à la maison** |
| 48 | doesn't like my friends | n'aime pas mes amis | n'aime pas mes amis |
| 49 | thinks I can’t take care of myself | pense que je ne sais pas prendre soins de moi | pense que je ne sais pas prendre soins de moi |
| 50 | repects my point of view | respecte mon point de vue | respecte mon point de vue |
| 51 | I am very proud of him | Je suis très fière de lui | Je suis très fière de lui |
| 52 | is very proud of me | est très fier de moi | est très fier de moi |
| 53 | seems to avoid problems | semble éviter d'aborder les problèmes | semble éviter d'aborder les problèmes |
| 54 | seems to be independent and helpless | semble être dépendant et sans défense | J'ai l'impression que mon père a besoin d'aide et qu'il est sans défense** |
| 55 | I feel like a burden to him | J'ai l'impression d'être un fardeau pour lui | J'ai l'impression d'être un fardeau pour lui |
| 56 | won't let me live my life | ne me laisse pas vivre ma vie | ne me laisse pas vivre ma vie |
| 57 | likes me as a person | m'aime en tant que personne | m'aime en tant que personne |
| 58 | isn’t a strong person | n'est pas une personne solide | n'est pas une personne solide |
| 59 | encourages me to make my own decisions | m'encourage à prendre mes propres décisions | m'encourage à prendre mes propres décisions |
| 60 | accepts that we may disagree | accepte que nous puissions ne pas être du même avis | accepte que nous puissions ne pas être du même avis |
| 61 | wants me to succeed at what I choose | veut que je réussisse ce que j'entreprends | veut que je réussisse ce que j'entreprends |
| 62 | accepts our differences | accepte nos différences | accepte nos différences |

Legend : *: if the item belongs to the revised IPR version proposed by the authors.

**: if the item was adapted during the qualitative work process.
