# Supplementary material for: Short version of the Inventory of Parental Representations, a self-report for attachment assessment among adolescents
Source: BMC Psychiatry. 2023 Apr 1;23:221. doi: 10.1186/s12888-023-04704-0 (PMC10068148; doi:10.1186/s12888-023-04704-0)
Supplement: Supplementary file 2 — Additional file 2. Different versions of the Inventory of Parental Representations proposed by the authors. Table which present the different structures of the Inventory of Parental Representations depending on different studies. [file 12888_2023_4704_MOESM2_ESM.docx]

Appendix 2. Different versions of the Inventory of Parental Representations proposed by the authors.

| Study (year) | Number of items | Number of dimensions | Dimensions |
| --- | --- | --- | --- |
| Solow (2004) | 2*62 | 7 | Self/Object (1, 3, 5, 7, 23, 31, 37, 42, 51)  Protected/Repaired Object (2, 10, 11, 30, 33, 34, 38, 40, 45)  Intrusive/Possessive Object (14, 18, 19, 27, 32, 47, 48, 49, 56)  Narcissistic/Hostile Object (12, 13, 16, 17, 22, 24, 26, 35, 36)  Facilitating of Individuation Object (4, 6, 8, 39, 43, 44, 50, 57, 59, 60, 61, 62)  Weak/Dependent Object (20, 21, 25, 28, 29, 41, 42, 46, 53, 54)  Demanding/Disappointed Object (8, 9, 15, 52, 54, 55, 58) |
| Soliman (2006) | 2*62 | 6 | Self/Object (for mother: 1, 3, 4, 5, 7, 23, 34, 37, 42, 51, 60, 62 and for father: 1, 3, 4, 5, 7, 10, 11, 23, 31, 36, 37, 42, 51)  Protected/Repaired (for mother: 2, 30, 33, 40, 45 and for father: 2, 30, 33, 38, 40, 45)  Intrusive/Possessive (for mother: 6, 8, 9, 14, 15, 18, 19, 25, 26, 27, 32, 43, 47, 48, 49, 56, 59 and for father: 9, 14, 15, 18, 19, 25, 26, 27, 32, 47, 48, 49, 56)  Narcissistic/Hostile (for mother: 11, 12, 13, 16, 17, 22, 24, 35, 36, 38, 41 and for father: 12, 13, 16, 17, 22, 24, 25, 35, 55)  Facilitating of Individuation (for mother: 10, 31, 39, 44, 50, 52, 57, 61 and for father: 6, 8, 39, 43, 44, 50, 52, 57, 60, 61, 62)  Weak/Dependent (for mother: 20, 21, 28, 29, 46, 53, 54, 55, 58 and for father: 20, 21, 28, 29, 41, 46, 53, 54, 58) |
| Shapiro (2016) | 2*19 | 5 | Narcissistic/Hostile (12, 16, 22, 24)  Protected/Repaired (34, 38, 40, 45, 52)  Intrusive/Possessive (18, 27, 32, 47)  Weak/Dependent (20, 21, 29)  Self/Object (5, 7, 23) |
