# Supplementary material for: Short version of the Inventory of Parental Representations, a self-report for attachment assessment among adolescents
Source: BMC Psychiatry. 2023 Apr 1;23:221. doi: 10.1186/s12888-023-04704-0 (PMC10068148; doi:10.1186/s12888-023-04704-0)
Supplement: Supplementary file 3 — Additional file 3. Paternal short version of the Inventory of Parental Representations: The Short IPRF. Presentation of the new questionnaire: The Short Inventory of Representations for Fathers (in French). [file 12888_2023_4704_MOESM3_ESM.docx]

Additional file 3. Paternal short version of the Inventory of Parental Representations: the Short-IPRF.

| Item | Nouvelle échelle Père | Item in original version | Dimension |
| --- | --- | --- | --- |
| 1 | me laisse faire mes propres choix | 6 | 2 |
| 2 | fait des choses pour m'humilier | 17 | 5 |
| 3 | ne me laisse aucun espace | 18 | 4 |
| 4 | se laisse marcher dessus | 21 | 1 |
| 5 | pense qu'il a toujours raison | 26 | 3 |
| 6 | ne peut pas me tenir tête | 28 | 1 |
| 7 | ne me laisse aucune intimité | 32 | 4 |
| 8 | entre en compétition avec moi | 35 | 5 |
| 9 | ne réagit pas quand j'ai des ennuis | 41 | 6 |
| 10 | me fait confiance | 44 | 2 |
| 11 | ne peut pas m'arrêter | 46 | 1 |
| 12 | pense que je ne sais pas prendre soins de moi | 49 | 4 |
| 13 | respecte mon point de vue | 50 | 3 |
| 14 | m'encourage à prendre mes propres décisions | 59 | 2 |
| 15 | accepte que nous puissions ne pas être du même avis | 60 | 3 |

*Legend :*

Dimension 1 : Reliability, dimension 2 : Autonomy, dimension 3 : Respect, dimension 4 : Intrusion, dimension 5 : Aggression, dimension 6 : Availability.
