# Supplementary material for: Short version of the Inventory of Parental Representations, a self-report for attachment assessment among adolescents
Source: BMC Psychiatry. 2023 Apr 1;23:221. doi: 10.1186/s12888-023-04704-0 (PMC10068148; doi:10.1186/s12888-023-04704-0)
Supplement: Supplementary file 4 — Additional file 4. Maternal short version of the Inventory of Parental Representations: The Short IPRM. Presentation of the new questionnaire: The Short Inventory of Representations for Mothers (in French). [file 12888_2023_4704_MOESM4_ESM.docx]

Additional file 4. Maternal short version of the Inventory of Parental Representations : the Short-IPRM.

| Item | Nouvelle échelle Mère | Item in original version | Dimension |
| --- | --- | --- | --- |
| 1 | m'aide à faire les choses que je veux faire | 4 | 2 |
| 2 | me laisse faire mes propres choix | 6 | 2 |
| 3 | fait des choses pour m'humilier | 17 | 5 |
| 4 | ne me laisse aucun espace | 18 | 4 |
| 5 | Nous nous comprenons parfaitement | 23 | 3 |
| 6 | ne me laisse aucune intimité | 32 | 4 |
| 7 | entre en compétition avec moi | 35 | 5 |
| 8 | est toujours là quand j'ai besoin d'elle | 37 | 6 |
| 9 | ne réagit pas quand j'ai des ennuis | 41 | 6 |
| 10 | accepte mes décisions | 43 | 3 |
| 11 | me fait confiance | 44 | 2 |
| 12 | respecte mon point de vue | 50 | 3 |
| 13 | ne me laisse pas vivre ma vie | 56 | 4 |
| 14 | m'encourage à prendre mes propres décisions | 59 | 2 |
| 15 | accepte que nous puissions ne pas être du même avis | 60 | 3 |
| 16 | accepte nos différences | 62 | 3 |

*Legend:*

Dimension 1: Reliability, dimension 2: Autonomy, dimension 3: Respect, dimension 4: Intrusion, dimension 5: Aggression, dimension 6: Availability.
